# Dr. Prichard on Epidemic Fever

**Published:** 1820-12-01

**Authors:** 


*A History of the Epidemic Fever which prevailed in Bristol, during the Years* 1817, 1818, *and* 1819, *founded on Reports of St. Peter's Hospital and the Bristol Infirmary.* By J. C. Prichard, M. D. &c. &c. Physician to St. Peter's Hospital and the Bristol Infirmary. Octavo, pp. 112, London, 1820.

Although Fever has subsided, and with it a great deal of the importance recently attached to all investigations of its etiology, pathology, and treatment; yet, as it is not likely that we shall ever have a long immunity from those epidemic influences which spread the disease throughout society, it is the bounden duty of the journalist, in particular, to record every contribution to our stock of knowledge on this interesting subject, not only as an index to future enquirers, but as a depôt from whence authentic information may hereafter be quickly drawn, upon sudden emergencies.

Dr. Prichard has, for some years, been well known to the profession, and to the literary world in general, by his learned researches into the physical history of man; and therefore we perused this little volume with considerable interest, to learn the sentiments of a man, respecting whose judgment we entertained favourable prepossession. Although Dr. Prichard has not entered so fully into many points of pathology and practice as we could have wished; yet we have been much gratified with what he has thought proper to communicate.

A considerable portion of the volume is occupied with tabular views of the fever, as it shewed itself more prominently in this or that organ. The tables also exhibit the duration and event of the disease; with notes respecting its propagation, and any remarkable circumstance connected with particular cases. These documents are valuable and authentic, but must necessarily be passed over in this place, in order that we may make known Dr. Prichard's sentiments relative to the pathology and treatment of the late epidemic fever, to which points he has chiefly confined his remarks on the present occasion.

Dr. Prichard conceives that the near alliance of fever with "the disease which *accompanies* the inflammation of particular organs," is every day more acknowledged by practitioners. We think that no man can doubt the similarity of idiopathic and symptomatic fevers; but many will doubt, and we among the number, whether both these fevers be dependant on the same cause—local inflammation. Neither can we agree with Dr. Prichard that it is a mere matter of curiosity, since we know that important practical indications hinge on the distinction. And here we would beg leave to correct an error into which Dr. Prichard, in common with several others, has fallen—namely, that a certain class of physiologists "avers that the arteries are never capable of any action at all." We would ask Dr. Prichard to point out one single physiologist who maintains such a doctrine! And if, Dr. P. be incapable of shewing any author who has ever hinted such a doctrine, we think it ought to make him and others a little cautious of allusions to tenets which have no foundation but in imagination or misconception. All we can say is, that our reading has furnished us with no such statements. If Dr. P. alludes to the doctrines of Dr. Parry of Bath, we can safely assert that that distinguished physician maintained that the arteries not only possess a power of action, but that they are constantly exercising this power, so long as life remains, or they themselves continue free from ossification.

We agree with Dr. Prichard that "in that milder form of the disease which is termed simple fever, there is no proof of any local inflammation;" and we nearly coincide with him in his other position, that "fever is only dangerous when it gives rise to, or displays the symptoms of, visceral inflammation." 48. Perhaps, however, this sentiment should be a little qualified. We have seen fever destroy life, before any sign of reaction, and consequently inflammation, became developed. In such cases we could not say that it is *only* dangerous when it displays the characters of the phlegmasiæ.

Dr. P. observes that the blood, even in simple fever, often exhibits the inflammatory crust.

"The state of the secretions is similar to the condition they assume in the phlegmasiæ; the relief also which is produced by venesection, and by other evacuations, tends to prove that the disease is, in its nature, analogous to these distempers."

He properly remarks that the mildest fevers are ever liable to be converted into the severer forms, and to exhibit, both in the living and the dead body, unequivocal marks of inflammation in the brain, the lungs, the liver, stomach, or bowels. The following sentiments appear to be a slight modification of Dr. Clutterbuck's theory of fever.

"Simple fever most naturally and frequently degenerates into cephalic; the head is more or less affected with pains, and other disorders in both cases. The seat of these, in the more severe form, seems to be the membranes of the brain; and to the same parts, as well as to the coverings of the nerves, we may, with great probability, ascribe the pains in the head and limbs, which attend the first attack of almost every case of fever. Simple and cephalic fever may therefore be considered as the genuine forms of this disease, the attacks being milder in the first, and more severe in the second. The pneumonic, hepatic, gastric, enteritic, and rheumatic forms may be regarded as varieties." 49.

Dr. Ducamp of Paris, a young physician of great talent and observation, in a late critique on a pamphlet of M. Chomel's relative to fever, seems to think that Broussais, whose doctrine he defends generally, has erred in confining the seat of inflammation in fever to the mucous membranes of the primæ viæ.

"M. Broussais a fait dériver les symptômes ataxiques, comme les symptomes adynamiques, de l'inflammation du tube digestif. Je crois qu'il a eu tort, et qu'il a dans ce cas fait jouer un trop grand rôle aux sympathies. On l'accuse de voir des phlegmasies partout, je vais lui addresser un reproche qui paraîtra fort étrange, c'est de ne pas voir *assez des phlegmasies* dans les cas de fievre ataxique, et de considerer comme sympathiques des phénomènes idiopathiques dependant immediatement de l'inflammation du cerveau ou ces annexes."

This is coming very near the doctrine of Dr. Mills in this country, and by the bye, of Dr. Rush, long ago in America—a doctrine overlooked too long by the European writers.

We shall now collect our author's sentiments relative to the treatment.

In a considerable number of those patients affected with the simple and cephalic forms of the fever, as they presented themselves at St. Peter's Hospital, and the Bristol Infirmary, the disease was still in its first stage—namely, "the period of depressed vital action." This cold stage he has frequently seen protracted many days, "without any appearance of re-action," in vagrants and other paupers who had long been exposed to cold and want.

"Occasionally these sunk in spite of all efforts to restore warmth and animation; in other instances, reaction, when it at length took place, was extremely violent, and produced a high degree of inflammation in some of the viscera, speedily terminating in effusion, suppuration, or gangrene, and death." 51.

Here, then, it is evident that the doctrine which maintains the identity of fever and inflammation, would lead to bad practice. If fever be inflammation, it is as much so in the first, as in any other stage; and ought to demand blood-letting and other evacuations. But practice gives the negative to theory here, as in too many other instances.

The first measure which was adopted at the institutions in question, was to strip the patients, put them into the warm bath, and shave the hair off their heads. While in the bath, they were directed to be well rubbed, with the double view of cleansing the surface, and drawing the blood to the same. They were next rubbed dry, and put to bed, where a draught of warm thin gruel was given them. In some instances, where there was great exhaustion, a little wine was added to the gruel.

"In general there was enough strength remaining to sustain without peril the action of vomiting. When this appeared to be the state of the patient, a gentle emetic dose was given to him, as soon as he came out of the warm bath, consisting of the powder of ipecacuanha, with or without an addition of tartarized antimony. By these means the powers of life were generally roused, and the hot stage brought on, often in a mitigated form. In a few cases the emetic cut short the complaint." 52.

The foregoing description applies principally to the vagrants received into St. Peter's Hospital: the persons brought to the infirmary were commonly in a less abject plight. In those that were admitted before reaction had taken place, the means abovementioned were adopted.

"It was during the first accession of the hot fit, that the chief attempts were made to subdue the disease by breaking through the train of morbid actions."

The symptoms now were, of course, those of strong reaction—a full, bounding pulse—hot, dry skin—parched, white, furred tongue, soon changing to a browner hue, with intense thirst, and more or less nausea. "But the part chiefly affected, was the head; the carotid and temporal arteries pulsated strongly; a dull oppressive pain was felt across the temples, and often severe shooting pains through the head."

An average venesection of ten or fourteen ounces almost uniformly relieved this state of the disease. "A disposition to syncope supervened, sooner than it would have done in a person of the same age and constitution labouring under pneumonic inflammation of the ordinary character." This is a common phenomenon in most fevers, shewing that the disease is not essentially inflammation, but the impression of a morbid, and hitherto untangible cause on the constitution, followed by a reactive process, very frequently producing inflammatory action in the most predisposed organ or tissue.

When an approach to syncope took place, accompanied with vomiting, "a surprising degree of relief was, in almost every instance, experienced." When the symptoms returned, after a lapse of ten or twelve hours, venesection was repeated, once, twice, or even thrice. "In persons of weak habits, or much exhausted, and in children under ten years of age, instead of bleeding from the arm or temporal artery, leeches were applied to the head." This was done, even in the case of robust persons, when the local affection continued after general bleeding, &c. had been used. In co-operation with vascular depletion, other means of reducing arterial action were employed. The head was shaved, and wetted with cold water; the body was sponged with the same. Cold affusion did not prove very serviceable:—the constant application of wet clothes, or frequent sponging, was a more efficacious method of abstracting heat.

The medicines administered were chiefly purgatives. "Five, six, or eight grains of calomel, with a moderate proportion of jalap, when the stomach was not irritable, were first given; if there was nausea or sickness, the calomel was ordered alone, or in pills with the compound extract of colocynth. This dose was followed by a purgative draught of infusion of senna with sulphate of magnesia, which was repeated every third, fourth, or sixth hour, according to the effect produced. The doses of calomel were afterwards repeated, without the powder or extract; the patient was freely purged for several days, and a very open state of the bowels maintained through the whole course of the disease."

This was almost uniformly the treatment in cephalic or simple fever; and in almost every instance, where the constitution of the patient was not impaired by habits of intemperance, or chronic maladies, the disease was speedily disarmed of its violence and danger. In a great proportion of cases the fever was cut short, and where this did not happen, its influence on the constitution was so much reduced, that the symptoms gradually declined afterwards, and the patient became convalescent in eight or ten days. When, however, the disease had crept on slowly, or got a fast hold of the constitution, before medical means were put in force, "the attempts to interrupt its course were seldom found to be successful." The measures then adopted were, of course, to obviate symptoms.

In cases complicated with thoracic and abdominal inflammation, the treatment underwent such modification as the structure and function of the seat of lesion obviously indicated, and need not be detailed here. The most unmanageable forms of fever were those where the mucous membrane of the stomach or bowels was inflamed; the former attended frequently with cholera, the latter with diarrhœa or dysentery. Here blood-letting could not be carried to the same extent as in the other modifications of fever. When drawn, however, the blood exhibited the buffy coat. One venesection was generally sufficient, after which leeches were applied to the epigastrium, or other part of the abdomen affected. To these were added the warm bath and fomentations, with blisters, effervescing draughts, opium, &c.

"But a more effectual relief was generally obtained by a few doses of calomel, containing five or six grains, in pills, combined with some of the resinous cathartics, or followed by a dose of ol. ricini, or the infusion of senna, with sulphate of magnesia, and repeated at the intervals of six or eight hours." 62.

Where neglected cases had degenerated into real typhus, the treatment was nearly the same, excepting that our author seemed to depend a good deal on the specific as well as purgative effects of calomel.

"The immediate effect of calomel upon the excretions was considered as a most important result of its use, but it was not the only one which was deemed a favourable indication. When it affected the constitution in that way which is manifested by soreness of the gums, and a disposition to ptyalism, it was, in a majority of cases, found that a gradual remission of the most formidable symptoms had taken place; the secretions became more natural in appearance; the alvine discharge of a better colour; the skin softer, and more perspirable." 68.

Evacuations of blood from the bowels were not always a fatal symptom. Even in these cases wine and stimulants are generally improper, and the antiphlogistic treatment must be pursued. Dr. Prichard saw one case of profuse hæmorrhage from the intestines happening in the course of fever, where a few doses of the tinct. ferri muriatis (30 drops every hour) arrested the flow of blood. Dr. P. thinks, however, that the tinct. lyttæ is one of the most efficacious remedies in cases of internal hæmorrage. We have found opium and superacetate of lead the most powerful restrainers of internal hæmorrhages.

"The use of wine and stimuli were (was) many times tried in those cases which have been supposed to call for such remedies, but they were always given with the caution which an experiment required, and generally abandoned on being found to be prejudicial." 73.

Dr. Pritchard lends his testimony to that of the many others who believe that fevers arise sometimes from contagion, sometimes from an incognizable atmospheric influence; and that, in either case, they may be propagated afterwards, from person to person, under certain deficiencies of ventilation, &c. &c. or under strong predispositions. This, our readers well know, is the prevailing doctrine of the best informed of the present times.

We have now exhibited a pretty full analysis of Dr. Prichard's work, which is calculated to impress the reader with a very favourable idea of our author's talent for accurate observation and judicious discrimination.

